# Integrated analysis of the oral and intestinal microbiome and metabolome of elderly people with more than 26 original teeth: a pilot study

**DOI:** 10.3389/fmicb.2023.1233460

**Published:** 2023-10-12

**Authors:** Yuichiro Nishimoto, Felix Salim, Kazuma Yama, Kota Kumagai, Ryutaro Jo, Minori Harada, Yuki Maruyama, Yuto Aita, Narumi Fujii, Takuya Inokuchi, Ryosuke Kawamata, Misato Sako, Yuko Ichiba, Kota Tsutsumi, Mitsuo Kimura, Yuka Mori, Shinnosuke Murakami, Yasushi Kakizawa, Takashi Kumagai, Shinji Fukuda

**Affiliations:** ^1^Metagen Inc., Tsuruoka, Yamagata, Japan; ^2^Department of Life Science and Technology, Tokyo Institute of Technology, Tokyo, Japan; ^3^Research and Development Headquarters, Lion Corporation, Tokyo, Japan; ^4^Hiyoshi Oral Health Clinics, Sakata, Yamagata, Japan; ^5^Institute for Advanced Biosciences, Keio University, Tsuruoka, Yamagata, Japan; ^6^Laboratory for Regenerative Microbiology, Juntendo University Graduate School of Medicine, Tokyo, Japan; ^7^Gut Environmental Design Group, Kanagawa Institute of Industrial Science and Technology, Kawasaki, Kanagawa, Japan; ^8^Transborder Medical Research Center, University of Tsukuba, Tsukuba, Ibaraki, Japan

**Keywords:** oral microbiome, oral metabolome, gut microbiome, gut metabolome, oral-gut axis

## Abstract

Elderly subjects with more than 20 natural teeth have a higher healthy life expectancy than those with few or no teeth. The oral microbiome and its metabolome are associated with oral health, and they are also associated with systemic health via the oral-gut axis. Here, we analyzed the oral and gut microbiome and metabolome profiles of elderly subjects with more than 26 natural teeth. Salivary samples collected as mouth-rinsed water and fecal samples were obtained from 22 healthy individuals, 10 elderly individuals with more than 26 natural teeth and 24 subjects with periodontal disease. The oral microbiome and metabolome profiles of elderly individuals resembled those of subjects with periodontal disease, with the metabolome showing a more substantial differential abundance of components. Despite the distinct oral metabolome profiles, there was no differential abundance of components in the gut microbiome and metabolomes, except for enrichment of short-chain fatty acids in elderly subjects. Finally, to investigate the relationship between the oral and gut microbiome and metabolome, we analyzed bacterial coexistence in the oral cavity and gut and analyzed the correlation of metabolite levels between the oral cavity and gut. However, there were few associations between oral and gut for bacteria and metabolites in either elderly or healthy subjects. Overall, these results indicate distinct oral microbiome and metabolome profiles, as well as the lack of an oral-gut axis in elderly subjects with a high number of natural teeth.

## 1. Introduction

High numbers of natural teeth (more than 20) at an elderly age have been associated with both greater life expectancy and greater healthy life expectancy (Hu et al., 2015; Matsuyama et al., 2017), suggesting the importance of dental health and dental care for longevity and health. Dental health is affected by many factors, including the oral microbiome and metabolome profiles. Oral microbes such as *Streptococcus mutans*, *Porphyromonas gingivalis*, *Tannerella forsythia*, and *Aggregatibacter actinomycetemcomitans* have been associated with dental caries and periodontal diseases (Sedghi et al., 2021). Other oral microbes have also been associated with systemic diseases such as gastric (Sun et al., 2017) and lung cancers (Zhang et al., 2019). The role of the oral microbiome in systemic disease, especially gastrointestinal disease, can be explained by the oral-gut axis. The oral microbiome and its metabolites may translocate to the gastrointestinal tract, inducing dysbiosis and intestinal inflammation (Kitamoto et al., 2020). Despite their importance, there is limited knowledge of the oral microbiome and metabolome of elderly individuals with more than 20 natural teeth due to limited cohort availability. In this study, we performed an exploratory analysis of the oral and gut microbiomes and metabolomes of elderly subjects with high numbers of natural teeth. A previous study reported that the number of teeth is associated with the oral microbiome composition (Takeshita et al., 2016). Therefore, the focus of this study was to compare elderly individuals with little or no tooth loss with healthy individuals to understand the effect of aging on oral microbiome and metabolome, excluding the effect of the number of teeth. Specifically, this study was conducted on elderly people who had 26 original teeth and whose oral conditions were well controlled by long-term maintenance by dentists. Compared to those in adult healthy subjects, the oral microbiome constituents and metabolites that were enriched in the elderly individuals more closely resembled those enriched in subjects with periodontal disease, with the metabolites having a more pronounced enrichment in the elderly subjects than in the subjects with periodontal disease. However, comparison between the healthy and elderly subjects’ gut microbiomes and metabolomes showed minimal differences, except for enrichment of short-chain fatty acids (SCFAs) in the elderly subjects. Composition and correlation analyses of saliva and fecal sample similarity showed that there was minimal overlap between the oral and gut microbes and metabolites in our cohort. Overall, we found signs of oral microbe-associated periodontal disease progression in elderly subjects, which may be repressed by diligent dental healthcare, as indicated by minimal loss of teeth.

## 2. Results

### 2.1. Study design and sample overview

**Figure 1:**
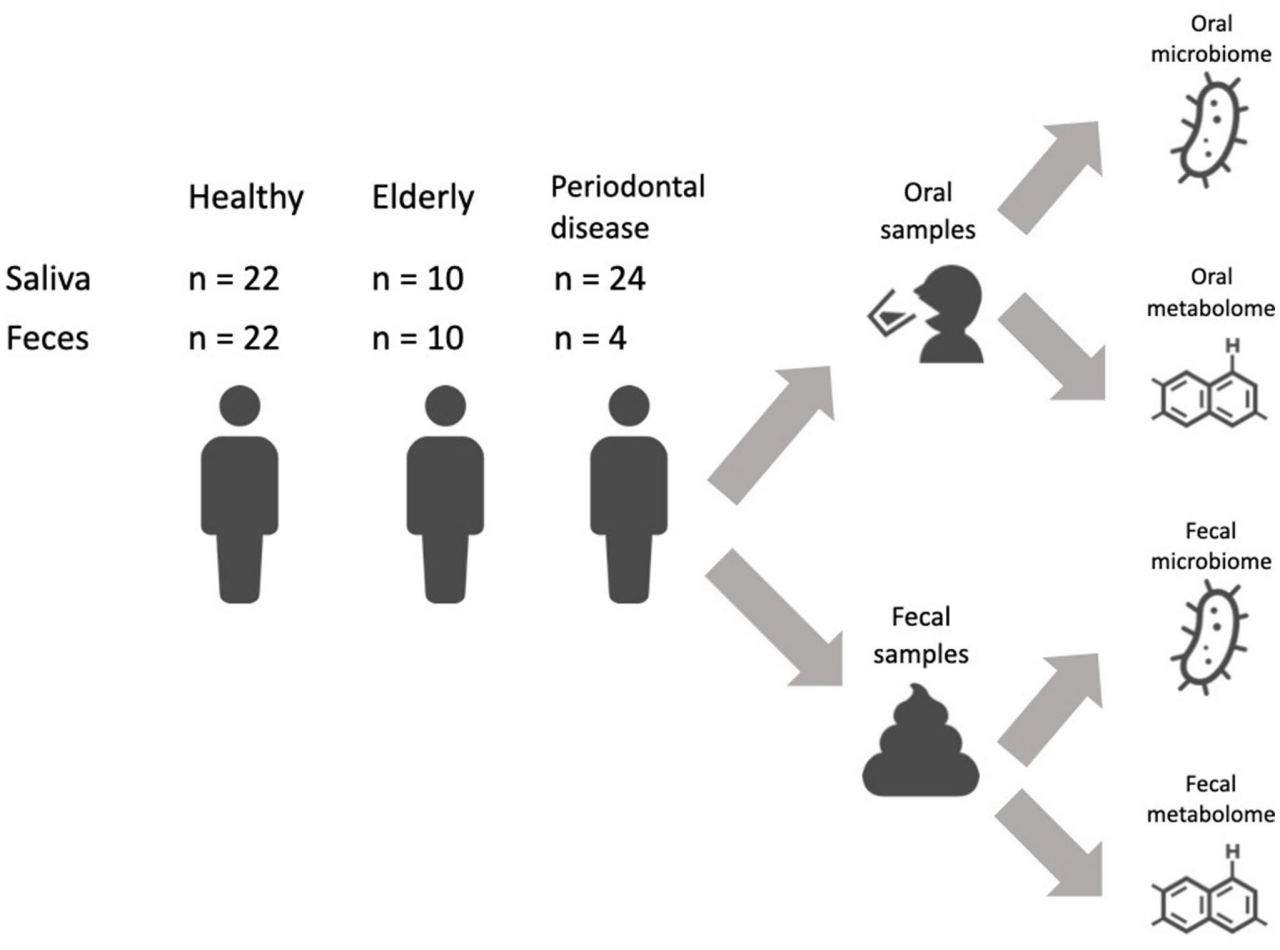
Study overview. Sampling design and sample size.

We recruited a Japanese cohort of 22 healthy individuals (age: 43.7 ± 11.8 years; 10 female and 12 male), 10 elderly individuals with more than 26 natural teeth (age: 83.4 ± 2.3 years; 5 female and 5 male) and 24 subjects with periodontal disease (age: 49.3 ± 12.0 years; 9 female and 15 male) (Figure 1; Supplementary Table 1). Subjects in the elderly group did not receive treatment for caries or periodontal disease in the prior 10 years, and oral conditions were well controlled by long-term maintenance by dentists. Subjects’ mouth-rinsed water, feces, anthropometric information (age and sex) and oral health indicator (number of teeth, rate of bleeding on probing (BOP) and rate of probing pocket depth (PPD) no less than 4 mm (PPD ≧4 mm) data were collected (Table 1). Elderly subjects had significantly higher BOP and PPD > 4 mm rates than healthy subjects but significantly lower rates than subjects with periodontal disease (Table 1).

**Table 1:** Basic information on the subjects.

	Healthy		Periodontal disease		Elderly	
	Mean	S.D.	Mean	S.D.	Mean	S.D.
Sample size	22		24		10	
Female/Male	10/12		9/15		5/5	
Age	43.7	11.8	49.3	12.0	83.4	2.3
Number of teeth	28.0	1.1	27.5	3.3	27.3	1.1
Rate of BOP	2.2	2.1	36.0	22.4	14.6	11.9
Rate of PPD no less than 4 mm	0.0	0.0	17.6	13.5	9.5	14.1

S.D., standard deviation; BOP, bleeding on probing; PPD, probing pocket depth.

### 2.2. The oral microbiome composition of the elderly group resembles the periodontal disease oral microbiome profile, but the metabolome profile is distinct

**Figure 2:**
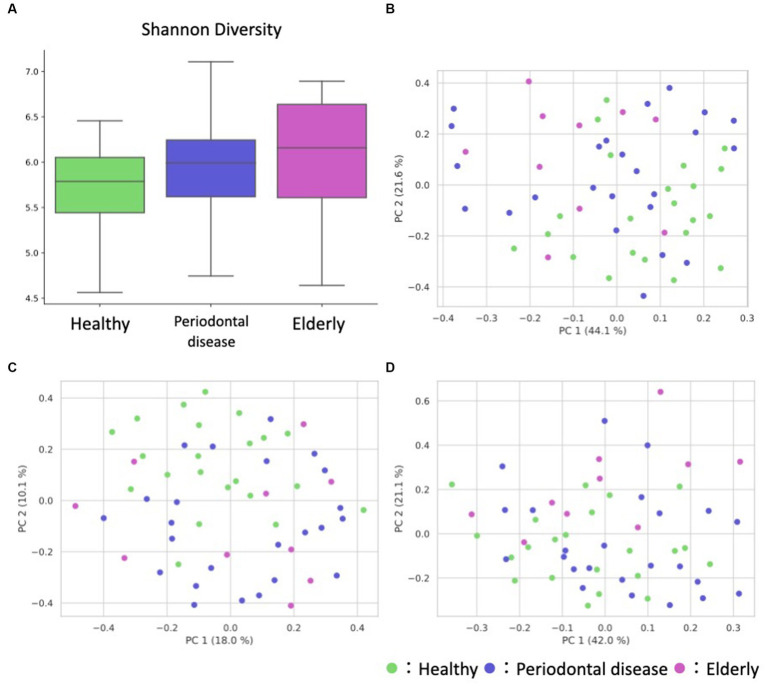
Overview of the oral sample microbiome and metabolome profiles. **(A)** Box plot of the alpha diversity indices (Shannon diversity) of oral microbiome profiles. **(B–D)** Oral ASV and metabolome profile PCoA annotated with the subject’s group principal coordinates. **(B)** ASV weighted UniFrac PCoA, **(C)** ASV unweighted UniFrac PCoA, **(D)** metabolome Bray–Curtis PCoA.

We conducted oral microbiome analysis by sequencing the amplified 16S rRNA gene. There was no significant difference in the alpha diversity of the oral microbiome among the healthy group, periodontal disease group, and elderly group (Figure 2A; Kruskal–Wallis test; *p* = 0.195). Principal coordinate analysis (PCoA) and permutational multivariate analysis of variance (PERMANOVA) of the oral microbiome were performed to determine whether the oral microbiome composition of elderly individuals was distinct from that of healthy adults or periodontal disease patients (Figures 2B–D). PCoA of weighted UniFrac distance showed distinct separation between healthy and elderly subjects but not between elderly individuals and subjects with periodontal disease. PERMANOVA showed that oral microbiome composition variance was significantly explained by the subject group for the elderly and healthy subjects but not for the elderly individuals and subjects with periodontal disease (Table 2; healthy vs. elderly R^2^ = 27.9%, Bonferroni corrected *p* = 0.015; periodontal disease vs. elderly R^2^ = 6.2%, Bonferroni corrected *p* = 1.000), supporting the PCoA results (Figures 2B,C).

**Table 2:** Beta diversity analysis of the oral microbiome and metabolome.

		Healthy vs. Elderly	Healthy vs. Periodontal disease	Elderly vs. Periodontal disease
Microbiome (weighted UniFrac)	*R^2^* score	0.279	0.144	0.062
	*p* value	0.015	0.036	1.000
Microbiome (unweighted UniFrac)	*R^2^* score	0.130	0.133	0.069
	*p* value	0.024	0.003	0.708
Metabolome (Bray–Curtis)	*R^2^* score	0.298	0.064	0.216
	*p* value	0.006	0.498	0.015

The numbers represent the *R*^2^ score and p value from the PERMANOVA result. The *p* value is the Bonferroni-corrected *p* value.

PCoA and PERMANOVA of the Bray–Curtis dissimilarity of the oral metabolome showed that the oral metabolome profiles of the elderly subjects were different from those of the individuals in the other groups (Figure 2D). PERMANOVA showed that the grouping significantly explained the oral metabolome composition variance for the healthy vs. elderly and elderly vs. periodontal disease groups (Table 2; healthy vs. elderly R^2^ = 29.8%, Bonferroni corrected *p* = 0.006; periodontal disease vs. elderly R^2^ = 21.6%, Bonferroni corrected *p* = 0.015).

**Figure 3:**
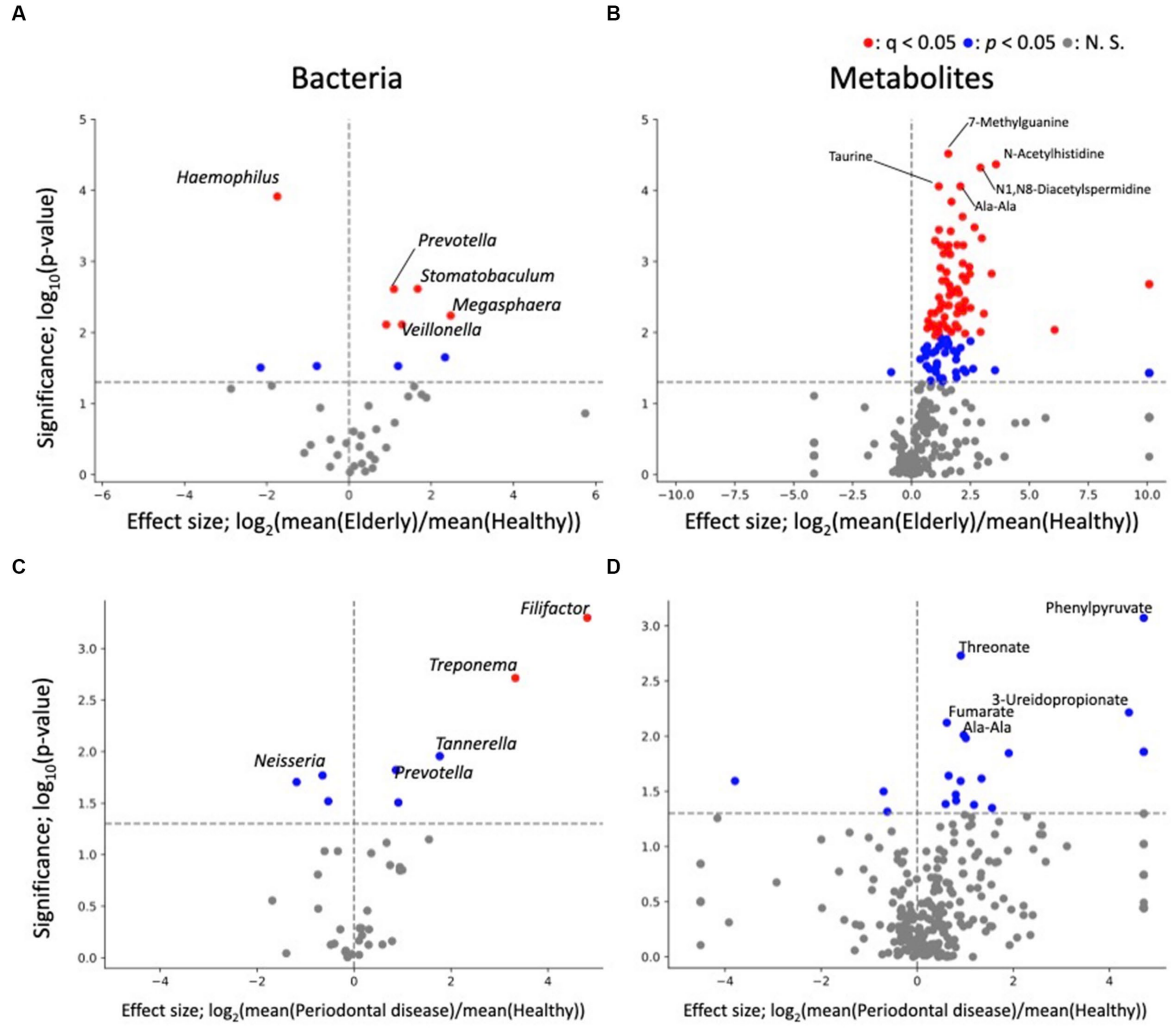
Differential abundance analysis of each oral microbe and metabolite. **(A,B)** Volcano plots of healthy vs. elderly group features; **(A)** genus; **(B)** metabolite. **(C,D)** Volcano plots of healthy vs. periodontal disease group features; **(C)** genus; **(D)** metabolite. The x-axis represents the effect size (logarithm of the fold change), and the y-axis represents the logarithm of the Wilcoxon rank sum test *p* value, based on comparison with the healthy group. Labels indicate the top 5 significant features.

As PERMANOVA revealed a distinct elderly oral microbiome and metabolome, differential abundance analysis was performed to identify oral microbiome and metabolome features that were associated with the elderly individuals (Figure 3). Genus level analysis results revealed 10 and 8 genera with significant differences in relative abundance (*p* < 0.05) from healthy vs. elderly (Figure 3A) and healthy vs. periodontal disease group comparisons (Figure 3C), with 5 genera showing significant differences in abundance in both comparisons. Among them, *Neisseria*, *Haemophilus*, and *Bergeyella* were enriched in the healthy group, and *Prevotella* and *Megasphaera* were depleted in the healthy group. False discovery rate (FDR) corrections (q < 0.05) reduced the significant features to 6 and 2 genera (enriched in the elderly group: *Prevotella*, *Veillonella*, *Oribacterium*, *Stomatobaculum* and *Megasphaera*; depleted in the elderly group: *Haemophilus*; enriched in the periodontal disease group: *Treponema* and *Filifactor*). Some features were observed in the elderly group, which may be influenced by the higher periodontal disease-related score in that group. Therefore, analysis of covariance (ANCOVA) between the periodontal disease and elderly groups was performed using the periodontal disease score (rate of PPD no less than 4 mm) as a confounding factor (Supplementary Table 2). The results showed that three microbes significantly more prevalent in the elderly group than in the healthy group were also significantly enriched by ANCOVA (*Oribacterium*, *Stomatobaculum*, and *Selenomonas*), and one of those less prevalent in the healthy group was also significantly less prevalent according to ANCOVA (*Haemophilus*).

The differential abundance analysis for each metabolite revealed significant differences in the levels of 114 metabolites between the healthy and elderly groups (Supplementary Table 2; Figure 3B) and in the levels of 20 metabolites between the healthy and periodontal disease groups (enriched in periodontal disease group; 2-hydroxy-4-methylpentanoate, 2-hydroxyglutarate + citramalate, 3-ureidopropionate, 4-hydroxymandelate, agmatine, Ala-Ala, CMP, fumarate, His, N1,N8-diacetylspermidine, octylamine, Phe, Phe-Phe, phenylpyruvate, threonate, urocanate, o-succinylhomoserine; depleted in the periodontal disease group; biotin, desthiobiotin, isobutylamine; Figure 3D), with 10 overlapping metabolites (2-hydroxy-4-methylpentanoate, 2-hydroxyglutarate + citramalate, agmatine, Ala-Ala, His, N1,N8-diacetylspermidine, Phe, Phe-Phe, threonate, urocanate; Supplementary Table 2). All 10 overlapping metabolites increased in abundance compared to that in the healthy group. After FDR correction, there were only 70 significant differences between the healthy and elderly groups. The oral metabolites of elderly individuals and subjects with periodontal disease showed partially similar enrichment patterns compared to those of healthy subjects. Except one metabolite (ethanolamine phosphate), 113 metabolites were significantly enriched, indicating that many metabolites were enriched in the elderly group (Supplementary Table 2). For example, oral SCFAs, such as butyrate and propionate, were enriched in both elderly individuals and subjects with periodontal disease compared to those in healthy subjects, but this enrichment was not significant in subjects with periodontal disease. As for bacteria, ANCOVA was performed using the periodontal disease score as a confounding factor. The results showed that for 82 metabolites, there was a significant increase in abundance after adjusting for periodontal disease score (Supplementary Table 2; *p* < 0.05). This indicates that many metabolites are enriched in the elderly group due to factors beyond the effects of periodontal disease.

### 2.3. The gut microbiome and metabolome are stable across the healthy and elderly groups

**Figure 4:**
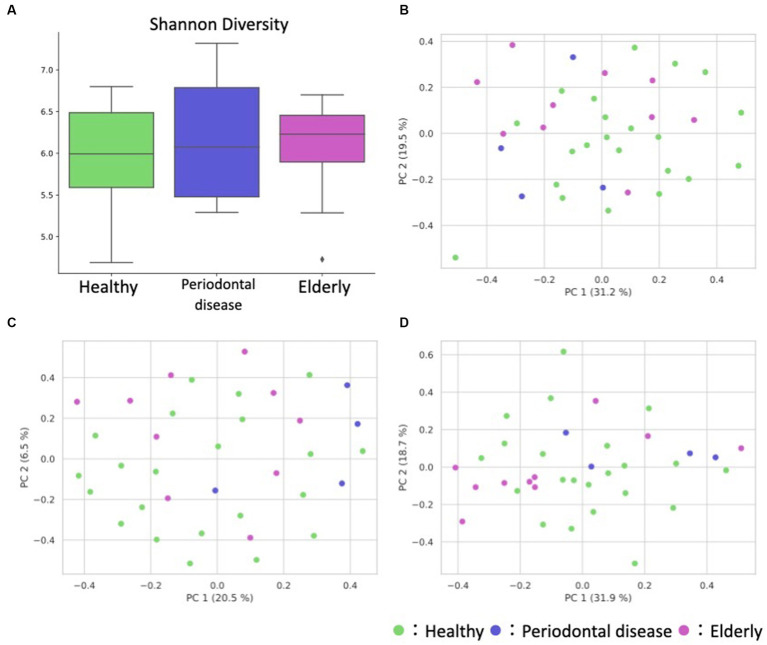
Overview of the gut microbiome and metabolome profiles. **(A)** Box plot of the alpha diversity indices (Shannon diversity) of gut microbiome profiles. **(B–D)** Fecal ASV and metabolite PCoA annotated with the subject’s group principal coordinates. **(B)** ASV weighted UniFrac PCoA, **(C)** ASV unweighted UniFrac PCoA, **(D)** metabolite Bray–Curtis PCoA.

We analyzed the gut microbiome and metabolome as well as oral samples. There was no significant difference in the alpha diversity of the gut microbiome among the healthy group, periodontal disease group, and elderly group (Figure 4A; Kruskal–Wallis test; *p* = 0.834). PCoA and PERMANOVA were also performed on the gut microbiome weighted/unweighted UniFrac distance and metabolome Bray–Curtis distance profiles of the healthy and elderly groups (Figures 4B–D; Table 3). Periodontal disease subjects were excluded from the PERMANOVA due to insufficient fecal samples (n = 4). PERMANOVA of the gut metabolome composition of the elderly and healthy subjects showed significant differences, but not for the gut microbiome (Table 3).

**Table 3:** Beta diversity analysis of the fecal microbiome and metabolome.

	*R^2^* score	*p* value
Microbiome (weighted UniFrac)	0.097	0.119
Microbiome (unweighted UniFrac)	0.094	0.051
Metabolome (Bray–Curtis)	0.126	0.043

PERMANOVA was performed for the healthy (*n* = 22) and elderly (*n* = 10) groups. The periodontal disease group was excluded from this analysis due to the small sample size.

**Figure 5:**
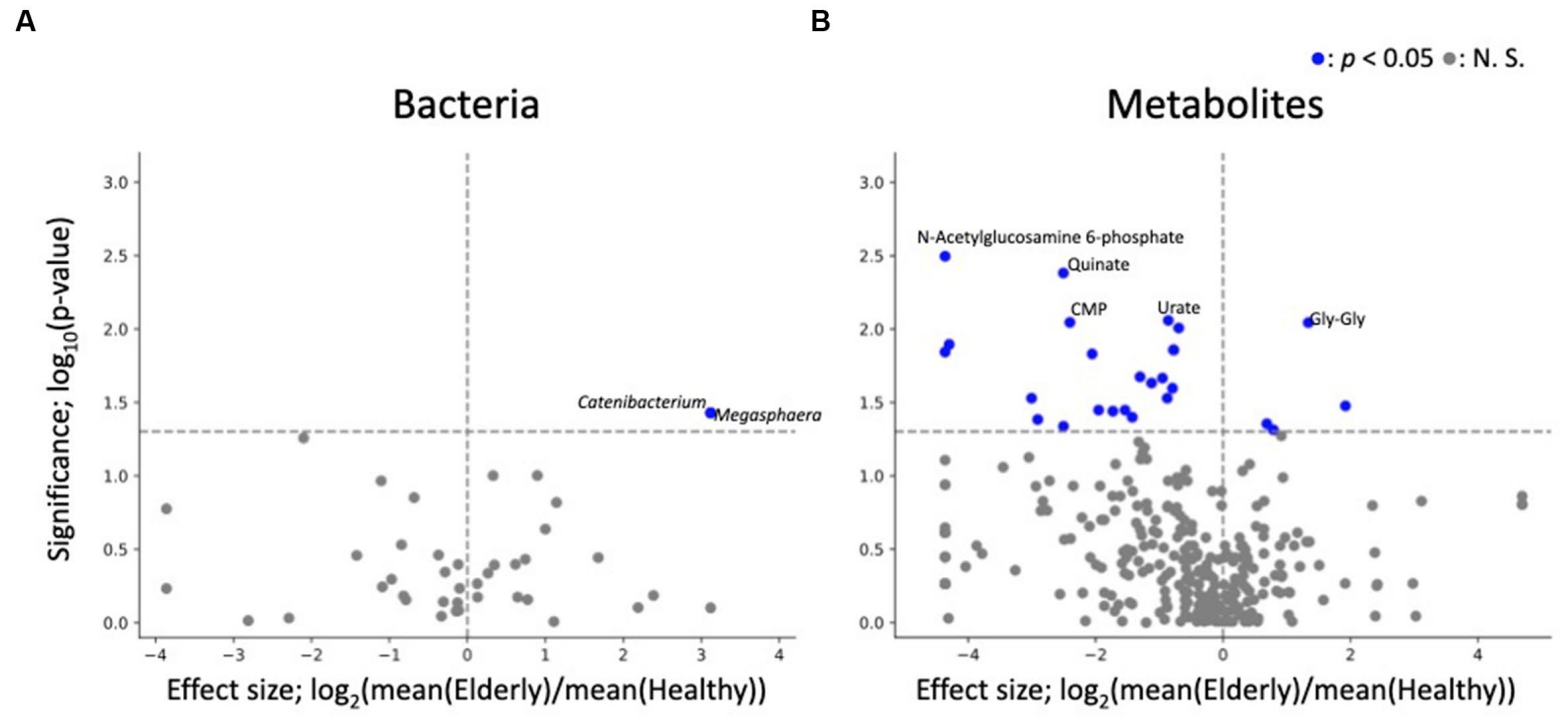
Differential abundance analysis of each fecal microbe and metabolite. **(A,B)** Volcano plots of fecal microbes and metabolites (healthy vs. elderly). Each dot represents a feature: **(A)** genus, **(B)** metabolites. The x-axis represents the effect size (logarithm of the fold change), and the y-axis represents the logarithm of the Wilcoxon rank sum test *p* value, based on comparison with the healthy group. Labels indicate the top 5 significant features.

Similar to the compositional analysis results, differential abundance analysis between the gut microbiome and metabolome of elderly and healthy subjects showed minimal differences, with no microbes or metabolites showing significant differences in abundance after FDR correction. Before FDR correction, two genera (*Megasphaera* and *Catenibacterium*) and 4 metabolites (Gly-Gly, 10-hydroxydecanoate, propionate, and butyrate) were enriched in the elderly group (Supplementary Table 3; Figure 5).

### 2.4. Minimal oral-gut axis in the healthy and elderly groups

Oral-gut transmission of microbes, especially in patients with gastrointestinal diseases, has been indicated by several studies (Schmidt et al., 2019; Kitamoto et al., 2020; Park et al., 2021), including reports demonstrating higher oral-gut microbiome similarity in elderly subjects than in healthy adults (Iwauchi et al., 2019). Therefore, to identify putative oral-gut transmission in our cohort, oral-gut association analyses were performed on the subjects’ oral and gut microbiomes and metabolomes.

**Figure 6:**
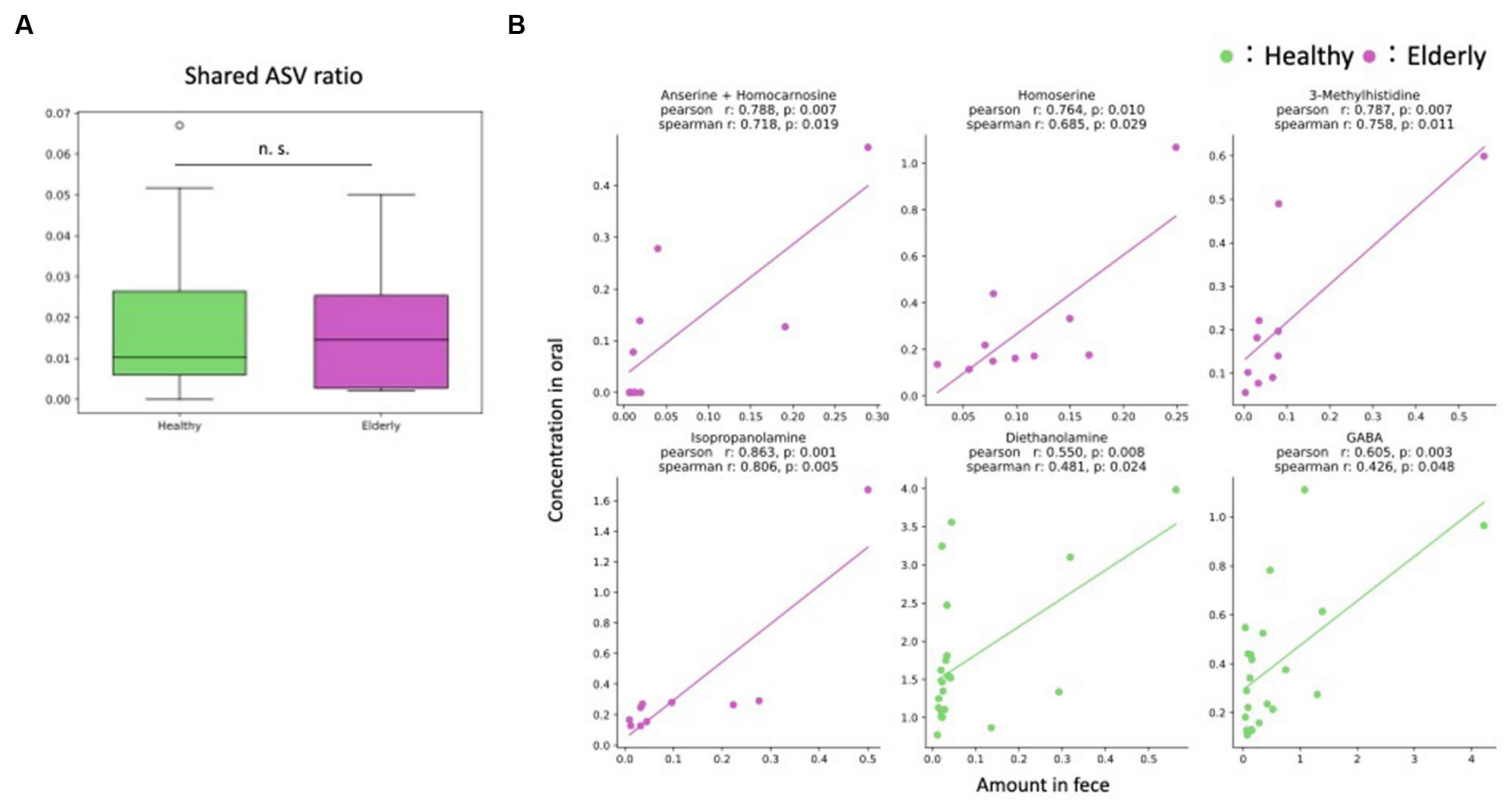
Oral-gut axis. **(A)** The ratio of ASVs detected simultaneously in the oral and gut to the total number of ASVs in both the oral and gut microbiome was calculated for each individual (shared ASV ratio); there were no significant differences between the healthy and elderly groups (Wilcoxon rank sum test). **(B)** Features that showed positive significant correlations in two indices, the Pearson and Spearman correlation coefficients. n. s., not significant; ASV, amplicon sequence variant.

First, we examined how many of the same amplicon sequence variants (ASVs) were detected in both the oral cavity and gut as an indicator of potential oral-gut transmission. To exclude the effect of the number of ASVs for each individual, the ratio of ASVs detected simultaneously in the oral and gut to the total number of ASVs in both the oral and gut microbiome was calculated for each individual (referred to as “shared ASV ratio”). As the however, only a few ratio of ASVs were detected in both the oral cavity and gut, indicating minimal oral-gut transmission (Figure 6A; Supplementary Table 4). In addition, we compared shared ASV ratio between the healthy and elderly groups, but no significant difference was detected (Wilcoxon rank-sum test; *p* = 0.807).

Next, correlation analysis was performed on metabolites that were detected in both the oral cavity and gut to further validate putative oral-gut transmission. Since most microbes were detected in only one of the two environments, correlation analysis was performed only for metabolites. Correlations were observed for some metabolites for each group. No metabolites showed significant correlations in both groups, but positive correlations were found for four metabolites in the elderly group (anserine + homocarnosine, homoserine, 3-methylhistidine and isopropanolamine) and for two metabolites in the healthy group (diethanolamine and gamma-amino butyric acid (GABA)) (Figure 6B; Supplementary Table 5).

## 3. Discussion

Compositional analysis of the oral microbiome and metabolome of elderly subjects with a high number of natural teeth (more than 26) revealed distinct compositions compared to those of healthy adult subjects, and the compositions more closely resembled those of subjects with periodontal disease. This observation was further supported by differential abundance analysis results, in which microbes and metabolites had more similar enrichment trends in elderly individuals and subjects with periodontal disease than healthy subjects. The similarity of oral microbes and metabolites between elderly individuals and subjects with periodontal disease may be associated with oral health indicators (i.e., BOP and PPD), as the elderly subjects had higher BOP and PPD ≧4 mm rates than the healthy subjects, although the BOP and PPD ≧4mm rates were still lower than those of subjects with periodontal disease. Therefore, the oral features of the elderly individuals found in this study may not be derived from periodontal disease-related events.

Differential abundance analysis of oral microbes revealed 2 significantly enriched genus (*Megasphaera* and *Prevotella*) and 3 significantly depleted genus (*Bergeyella*, *Haemophilus* and *Neisseria*) in both elderly individuals and subjects with periodontal disease compared to those in healthy subjects. Depletion of *Neisseria* were consistent with previous reports of the oral microbes of subjects with periodontal disease (Ikeda et al., 2020; Cai et al., 2021). Differential abundance analysis of oral metabolites showed that most significant differences were found in the healthy vs. elderly comparison, in which 70 metabolites were significantly enriched in elderly subjects after FDR correction. In comparison, there were 20 metabolites showing significantly differential abundance in subjects with periodontal disease, but none of the differences remained significant after FDR correction. Several metabolites enriched in elderly subjects, such as propionate, butyrate and 5-aminovalerate, were associated with periodontal disease. High levels of SCFAs such as propionate and butyrate have been associated with periodontal disease (Na et al., 2021), which indicates that changes in the levels of oral metabolites associated with aging resemble those associated with periodontal disease. Our results suggest that even with minimal tooth loss, oral metabolite profiles in elderly individuals change into periodontal disease-associated metabolite profiles.

Observations of subjects’ gut microbiome and metabolome composition revealed minimal differences between elderly and healthy subjects’ gut microbiome profiles but somewhat distinct metabolome profiles. Most metabolites were more abundant in the healthy adult gut, with Gly-Gly, 10-hydroxydecanoate, propionate and butyrate being exceptions. Of these, SCFAs (propionate and butyrate) play key roles in regulating colon physiology and changing the intestinal environment (Cong et al., 2022). Previous studies have reported that having fewer teeth results in lower dietary intake of fiber, a source of SCFAs (Nakamura et al., 2019). It is possible that the presence of teeth helps elderly individuals maintain a healthy diet high in calories. The lack of fecal samples in the periodontal disease group is also a major limitation in the interpretation of this study. The results of the microbiome PCoA when using unweighted UniFrac distance show that the periodontal disease group is relatively clustered. Therefore, the gut microbiome of the periodontal group may have some unique features. However, these results are inconclusive, and further samples are needed.

There have been several reports on oral-gut microbiome transmission and its effects on host health (Schmidt et al., 2019; Kitamoto et al., 2020; Park et al., 2021). We observed minimal overlap between the oral and gut microbiome. This result indicated the lack of oral-gut microbiome transmission within our cohort. Metabolite correlation analysis showed only four and two positive significant correlations in the elderly and healthy groups, respectively. However, of the six metabolites with significant positive correlations (anserine + homocarnosine, homoserine, 3-methylhistidine, isopropanolamine, diethanolamine and GABA), none has been reported to be involved in the oral-gut axis. Overall, we found minimal evidence of an oral-gut axis in our cohort of healthy subjects and elderly individuals with high numbers of natural teeth. The results were contrary to previous studies, which suggested oral-gut bacterial transmission in elderly individuals (Iwauchi et al., 2019). In that previous study, the elderly subjects had a low number of teeth (mean: 18.8). In another previous study, it was reported that oral bacteria increase in abundance in the gut when drugs that inhibit gastric function, such as proton pump inhibitors, are ingested (Imhann et al., 2016). The presence of teeth suggests that gastrointestinal function is not weakened in the elderly individuals and that oral-gut transmission may not have occurred as a result. These results might also be explained by the lack of gastrointestinal disease and minimal tooth loss in our cohort, in accordance with a previous study showing minimal overlap between oral and gut microbes in healthy adults (Rashidi et al., 2021).

Our study has some limitations, such as the lack of dietary data and small sample size. In addition, although the aim of the study was to characterize the bacteria and metabolites of elderly subjects with more than 26 natural teeth, the age of the control, healthy group, was different from that of the elderly group, which may have accentuated the age-related characteristics of the elderly group. Therefore, a study with larger, and age-matched cohort is essential to validate our results. In addition, the paucity of gut microbiome and metabolome data in the periodontal disease group poses a major limitation to the interpretation of the gut-oral axis analysis in this study. In particular, the potential of the existence of a gut-oral axis for the periodontal disease group requires further study.

In summary, we found that elderly subjects with a high number of teeth had distinct oral microbiome and metabolome profiles compared to those of healthy subjects. The oral microbiome and metabolome profiles of the elderly subjects resembled those of the subjects with periodontal disease, showing a similar enrichment pattern with a more obvious metabolite disruption than even that in subjects with periodontal disease. However, the gut microbiome and metabolome did not show such a distinct composition, with only a few microbes or metabolites with differential abundances between healthy and elderly subjects. Overall, our observation suggests that age-related changes occur in the oral environment even in the absence of tooth loss. These changes may induce periodontal disease progression but may be counteracted by proper dental care.

## 4. Methods

### 4.1. Ethics statement

This trial was approved by the clinical trial ethics review committee of the Chiyoda Paramedical Care Clinic and reported to https://www.umin.ac.jp/ (identifier: UMIN000031334). All participants understood the study objective and provided written informed consent, which we affirmed. All experiments were performed in accordance with approved guidelines.

### 4.2. Trial design and recruitment

Participants were recruited at the Hiyoshi Oral Health Clinics and grouped into healthy, periodontal disease and elderly groups based on the following criteria. The healthy group criteria were (a) between 20 and 79 years old, (b) all PPD less than 4 mm, (c) BOP tooth surface less than 10%, (d) no dental caries, (e) no denture or implants, (f) nonsmoker, (g) no orthodontic treatment and (h) no antibiotic consumption during the prior 6 months. The periodontal disease group criteria were as follows: (a) between 20 and 79 years old, (b) with bleeding from PPD ≧ 4 mm, (c) no carious pit, (d) no denture or implants, (e) nonsmoker, and (f) no antibiotic consumption during the prior 6 months. The elderly group criteria were (a) between 80 and 100 years old, (b) more than 26 original teeth, and (c) did not receive treatment for caries or periodontal disease during the prior 10 years. All participants were instructed not to eat, drink, or brush their teeth for at least 1 h prior to sampling. The sample size for the elderly group was set to ten. Preliminary power calculations were not performed, as there were no reference studies. However, it was assumed to have a large effect size for the elderly group, and the sample size was set to ten in accordance with previous studies (Whitehead et al., 2016).

### 4.3. Sample collection

Participants’ oral samples were collected at Hiyoshi Oral Health Clinics, as previously described (Jo et al., 2019). Specifically, participants were instructed to wash their mouths with 3 mL of sterilized water, which was collected for microbiome and metabolite extraction. All samples were stored in a freezer within 10 min of collection and kept at −80°C until further processing.

Participants’ oral health conditions were examined after sample collection. The numbers of original, decayed, missing and filled teeth were recorded as indicators of participants’ caries history. PPD and BOP at four sites (mesiobuccal, distobuccal, mesiolingual, and distolingual) of all teeth were measured with a periodontal pocket probe as indicators of periodontal disease.

### 4.4. DNA and metabolite extraction from fecal and mouth-rinsed water samples

DNA extraction from mouth-rinsed water and fecal samples was performed as previously described (Jo et al., 2019; Hashimoto et al., 2023). PCR was conducted using universal primers (27Fmod and 338R) for 16S rRNA gene sequencing, as previously described. PCR was performed using Ex Taq polymerase (Takara Bio, Shiga, Japan) and approximately 20 ng of template DNA.

Thermal cycling was performed in a Veriti Thermal Cycler (Life Technologies Japan, Tokyo) with the following cycling conditions: initial denaturation at 96°C for 2 min, followed by 25 cycles of denaturation at 96°C for 30 s, annealing at 55°C for 45 s, and extension at 72°C for 1 min, with a final extension at 72°C for 10 min. PCR amplicons were purified using AMPure XP magnetic purification beads (Beckman Coulter, Brea, CA, USA) and quantified using a Quant-iT PicoGreen dsDNA Assay Kit (Life Technologies Japan). After quantification, mixed samples were prepared by pooling approximately equal amounts of each amplified DNA and sequenced using a MiSeq Reagent Kit V3 (300 bp × 2 cycles) and a MiSeq sequencer (Illumina, CA, USA) according to the manufacturer’s instructions.

The extraction of metabolites from mouth-rinsed water and fecal samples was also previously described (Maruyama et al., 2022). Frozen saliva samples were thawed and centrifuged at 13,000 × *g* for 5 min at 4°C. The supernatant was transferred to a 5-kDa-cutoff filter (Human Metabolome Technologies, Tsuruoka, Japan) to remove proteins of sizes greater than 5 kDa. Prior to CE-TOFMS analysis, a 45 μL aliquot of the filtrate was added to 5 μL of Milli-Q water containing reference compounds (200 mmol/L each of methionine sulfone, D-camphor-10-sulfonic acid, 3-aminopyrrolidine, and trimesic acid). CE-TOFMS-based metabolome profiling was performed using an Agilent 7,100 capillary electrophoresis system (Agilent Technologies, Waldbronn, Germany), an Agilent 6,224 TOF LC/MS system (Agilent Technologies, Santa Clara, CA), an Agilent 1,200 series isocratic HPLC pump, a G1603A Agilent CE-MS adapter kit, and a G1607A Agilent CE-electrospray ionization (ESI)-MS sprayer kit. In anionic metabolite analysis, the ESI sprayer was replaced with a platinum needle instead of an initial stainless-steel needle. Other conditions of the CE–ESI–MS sprayer were the same as received. The metabolome analysis conditions were the same as those described previously (Sugimoto et al., 2010). Data analysis was performed using the metabolome analysis software MasterHands as previously described (Hirayama et al., 2009).

### 4.5. Bioinformatics and statistical analysis

For 16S rRNA gene analysis, QIIME2 (version 2019.10) was used (Bolyen et al., 2019). In the analytical pipeline, adapters were trimmed by cutadapt (options: --p-front-f AGRGTTTGATYMTGGCTCAG --p-front-r TGCTGCCTCCCGTAGGAGT --p-discard-untrimmed) (Martin, 2011). Trimmed sequence data were processed by using the DADA2 pipeline for quality filtering and denoising (options: --p-trunc-len-f 275 --p-trunc-len-r 230) (Callahan et al., 2016). The filtered output sequences were assigned to taxa by using the “qiime feature-classifier classify-sklearn” command with the default parameters.

SILVA LTP (version 132) was deduplicated prior to classifier training. The identified duplicate sequences were compared to LTP_2020, and those not included in LTP_2020 were also removed from the SILVA LTP database used in this study. The deduplicated SILVA LTP was combined with the extended HOMD (eHOMD) 16S rRNA RefSeq (version 15.22) database and then processed by using the “qiime feature-classifier extract-reads” command (options: --p-f-primer AGRGTTTGATYMTGGCTCAG --p-r-primer TGCTGCCTCCCGTAGGAGT --p-min-length 150 --p-max-length 450) prior to classifier training. The taxon classifier was trained by using the “qiime feature-classifier fit-classifier-naive-bayes” command with the default parameters. UniFrac (weighted and unweighted) distance was calculated from rarified samples’ ASVs using the “qiime diversity core-metrics-phylogenetic” command (options: --sampling-depth 7,000). PERMANOVA was performed with scikit-bio. Other statistical analyses were performed with in-house Python scripts (version 3.7.6). For pairwise comparison of the abundances of microbes and metabolites, the Wilcoxon rank sum test [scipy version 1.5.2 (Virtanen et al., 2020)] with Benjamini–Hochberg FDR correction (statsmodels version 0.10.0) was used. For the comparison, microbial taxa with a mean relative abundance below 0.001 were excluded. When comparing the abundance of bacteria and metabolites between elderly and periodontal disease group, ANCOVA was performed after performing ranking (pingouin version 0.5.3). Rate of PPD no less than 4 mm was used as a confounding factor. In addition, metabolites detected in 50% of the saliva and fecal samples were included in the oral-gut metabolite correlation analysis.

## Data availability statement

The datasets presented in this study can be found in online repositories. The names of the repository/repositories and accession number(s) can be found in the NCBI repository under BioProject accession number PRJDB15683 in the DDBJ BioProject database.

## Ethics statement

The studies involving humans were approved by committee of the Chiyoda Paramedical Care Clinic. The studies were conducted in accordance with the local legislation and institutional requirements. The participants provided their written informed consent to participate in this study.

## Author contributions

TK and SF: conceptualization. YN: data curation. YN and FS: formal analysis and writing – original draft. KY, RJ, and SM: methodology. YN, FS, KY, KK, RJ, MH, YMa, YA, NF, TI, RK, MS, YI, KT, MK, YMo, SM, YK, TK, and SF: writing – review and editing. All authors contributed to the article and approved the submitted version.
